# Reconstruction of a chronically ruptured Achilles tendon using an internal brace: a case report

**DOI:** 10.1186/s13256-018-1610-6

**Published:** 2018-03-02

**Authors:** Shuichi Chida, Hitoshi Suzuki, Moto Kobayashi, Tsutomu Sakuraba, Hideji Kura, Naohisa Miyakoshi, Yoichi Shimada

**Affiliations:** 1 Department of Orthopedic Surgery, Kakunodate General Hospital, 3-Iwase Kakunodatemachi, Senboku, Akita 014-0394 Japan; 2 Department of Orthopedic Surgery, Hiraka General Hospital, 3-1 Maego aza Yatsuguchi, Yokote, Akita 013-8610 Japan; 3 Department of Orthopedic Surgery, Hitsujigaoka Hospital, 3-1-10 Aobamachi, Atsubetsuku, Sapporo, 004-0021 Japan; 4 Department of Orthopedic Surgery, Akita University Graduate School of Medicine, 1-1-1 Hondo, Akita, 010-8543 Japan

**Keywords:** Chronic rupturing of Achilles tendon, Achilles Midsubstance SpeedBridge Repair, Rehabilitation

## Abstract

**Background:**

We reconstructed a chronically ruptured Achilles tendon and the associated scar tissue using braided polyblend polyethylene sutures (FiberWire; Arthrex Inc.; Naples, FL, USA) and anchors.

**Case presentation:**

A 68-year-old Japanese man, who was being treated for right Achilles tendinosis, felt pain in his Achilles tendon when walking and started to find plantar flexion of his ankle joint difficult. As his symptoms persisted, he visited us after 4 weeks. Surgery and orthotic therapy were recommended, but he did not want to undergo these treatments. However, he began to find walking difficult and so underwent surgery 6 months after suffering the injury. The interior of the tendon was curetted, and the ruptured region was subjected to plication using the surrounding scar tissue. Using the percutaneous Achilles repair system (Arthrex Inc.), FiberWire sutures were inserted, and two skin incisions were made on the medial and lateral sides of his calcaneus in the region surrounding the Achilles tendon attachment. SutureLasso (Arthrex Inc.) was passed through, and the proximal FiberWire suture was relayed and fixed with 4.75-mm SwiveLock (Arthrex Inc.). After surgery, his foot was fixed in plaster at 20° plantar flexion of his ankle joint. The plaster was removed 1 week after surgery, and after-treatment was initiated with active dorsiflexion training. No orthosis was used after surgery. As of 16 postoperative months, no re-rupture had occurred.

**Conclusions:**

This method might allow post-treatment rehabilitation, and so on, to occur earlier, and, hence, could become an option for the reconstruction of chronically ruptured Achilles tendons.

## Background

We reconstructed a chronically ruptured Achilles tendon and the scar tissue using the Achilles Midsubstance SpeedBridge™ Repair (Arthrex Inc.; Naples, FL, USA) with braided polyblend polyethylene sutures (FiberWire®; Arthrex Inc.) and anchors.

## Case presentation

**Fig. 1:**
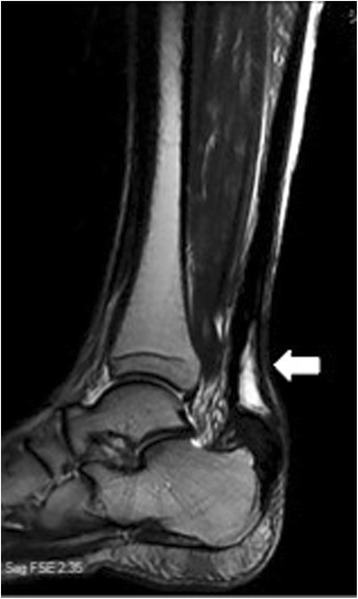
Preoperative magnetic resonance imaging. Parts of the Achilles tendon remained intact, but the tendon was extended (*arrow*)

A 68-year-old Japanese man, who was being treated for right Achilles tendinosis, felt pain in his Achilles tendon when walking and started to find plantar flexion of his ankle joint difficult. As his symptoms persisted, he visited us after 4 weeks. Pain and slight concavity were palpated in his Achilles tendon, and the Simmonds–Thompson squeezing test produced a positive result. A ruptured Achilles tendon was observed on magnetic resonance imaging (MRI). Surgery and orthotic therapy were recommended, but he did not want to undergo these treatments. However, he started to find walking difficult and underwent surgery 6 months after suffering the injury. At this point, the Simmonds–Thompson squeezing test produced a positive result. A palpable deficit in his Achilles tendon was identified on examination, and he could not raise his heel. The Japanese Society of Surgery of the Foot (JSSF) ankle/hindfoot scale [1, 2] was 53 points. On MRI, it was found that parts of the Achilles tendon were intact, but the tendon was elongated (Fig. 1).

**Fig. 2:**
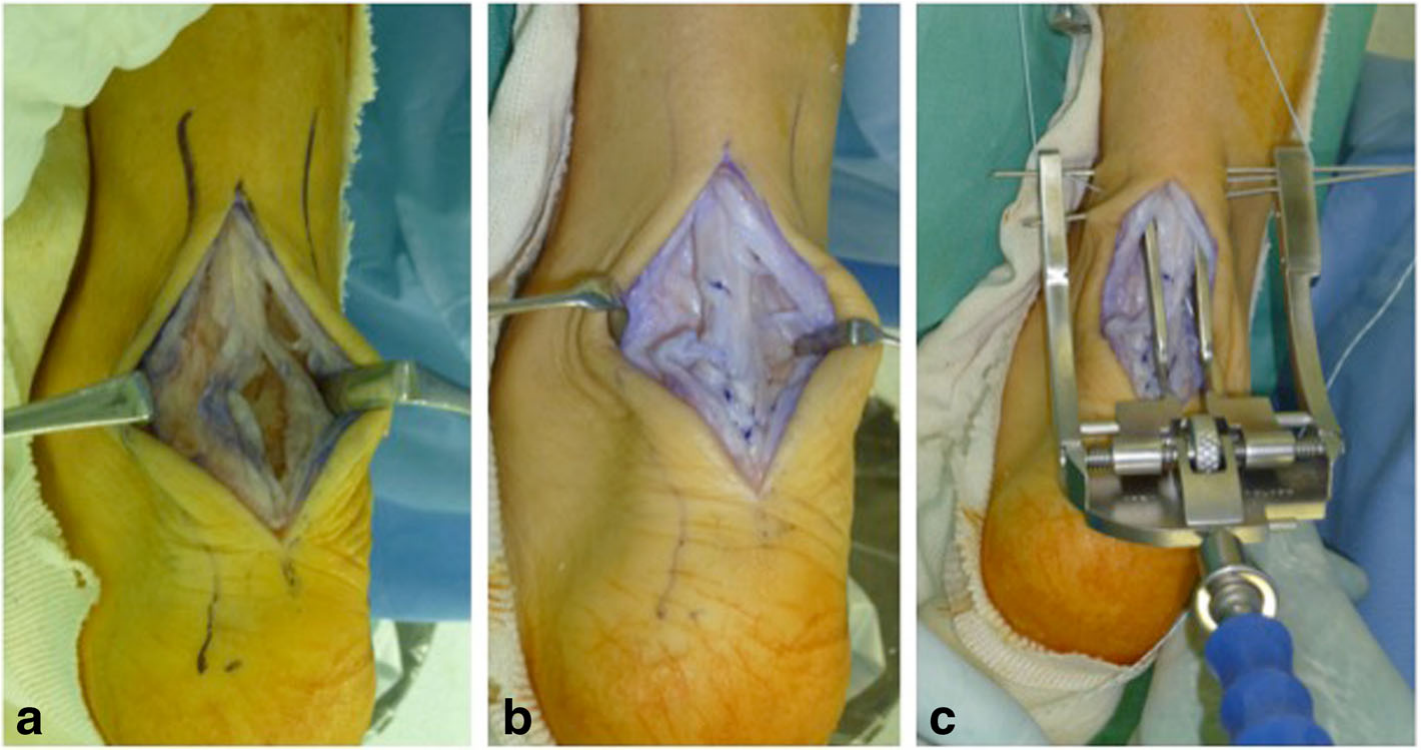
Intraoperative findings. **a** The ruptured region of the tendon had been cicatrized, and the interior of the tendon was hollow. **b** The ruptured region was subjected to plication using scar tissue. **c** Achilles Midsubstance SpeedBridge repair was performed concomitantly

During surgery, a 7-cm skin incision was made centering on the ruptured region of our patient’s tendon. The ruptured region had been cicatrized. When it was incised, it was found that the interior of his tendon had become hollow, and some yellow exudate was found within it (Fig. 2a). The interior of the ruptured region was curetted, and scar tissue around the ruptured region was plicated with 3-0 absorbable sutures (Fig. 2b). At this point, his ankle joint was able to undergo 20° plantar flexion; that is, his Achilles tendon exhibited favorable tension. Then, a percutaneous Achilles repair system (PARS; Arthrex Inc.) jig was inserted at a proximal site. The PARS needle guide pins were inserted in order, a FiberWire was passed through the jig, and two simple sutures and one locked suture were applied (Fig. 2c). Two skin incisions were made on the medial and lateral sides of his calcaneus in the region surrounding Achilles tendon attachment, SutureLasso™ (Arthrex Inc.) was passed through, and the proximal FiberWire suture was relayed. Two burr holes were prepared in his calcaneus, and fixation was achieved with 4.75-mm Bio-SwiveLock anchor (Arthrex Inc.). After surgery, his foot was put in a cast at 20° plantar flexion of his ankle joint.

**Fig. 3:**
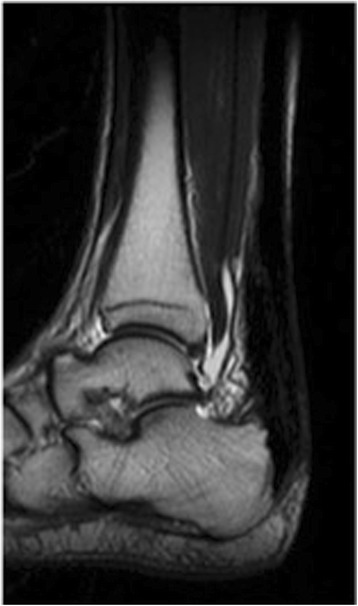
Magnetic resonance imaging scan obtained at 6 postoperative months. Continuity of the Achilles tendon was observed

The plaster was removed 1 week after surgery, and postoperative treatment was initiated with active dorsiflexion training. No orthosis was used after surgery; partial weight bearing was started after the acquisition of 0° dorsiflexion, and full weight bearing was initiated after 4 postoperative weeks. Our patient started jogging at 8 postoperative weeks and was permitted to play sports from 4 postoperative months. As of 16 postoperative months, no re-rupture had occurred, he was able to raise his bilateral heels and unilateral heel normally, and the JSSF ankle/hindfoot scale was 100 points. Continuity of the Achilles tendon was observed on MRI obtained at 6 postoperative months (Fig. 3).

## Discussion

During the reconstruction of a chronically ruptured Achilles tendon, cicatrectomy is performed, and after creating a fresh stump reconstruction is carried out via: (1) the Lindholm or Bosworth method using a gastrocnemius fascia flap, (2) V-Y plasty of the proximal stump, (3) a tendon transfer, or (4) the use of an artificial ligament [3–7]. Tendinous tissue regenerates after the repair of scar tissue stumps, and a reconstruction procedure involving scar tissue, in which the scar tissue was shortened by partially resecting it between the stumps and the stumps were sutured end-to-end, has been reported [8–10]. The direct repair of scar tissue without an allograft or autograft was found to be an effective treatment for chronically ruptured Achilles tendons. However, all treatment methods for chronically ruptured Achilles tendons require casting or orthotic therapy during the postoperative period.

Achilles Midsubstance SpeedBridge repair is a percutaneous suturing method that is applied to recently ruptured Achilles tendons. In this method, a specifically designed jig is inserted into the proximal stump through a small incision followed by FiberWire sutures. This procedure enables the appropriate guidance and locking of sutures. A FiberWire suture is then guided towards the calcaneus and fixed in place with an anchor. This is a minimally invasive suture method that results in stronger internal fixation than can be achieved using conventional Krackow sutures [11].

In the present case, a chronically ruptured Achilles tendon was subjected to plication using scar tissue, and an internal brace using the Achilles Midsubstance SpeedBridge repair was performed concomitantly, which resulted in strong internal fixation and allowed our patient to undergo early rehabilitation and to return to his previous activities without requiring an orthosis after surgery. This method might be useful for treating both recently and chronically ruptured Achilles tendons. However, we have only described the use of this method in one case, and so further study of this technique will be necessary in the future.

## Conclusion

For chronically ruptured Achilles tendons, an internal brace involving the direct repair of scar tissue can achieve strong internal fixation, which in turn facilitates early rehabilitation.

## Abbreviations

JSSF: Japanese Society of Surgery of the Foot
MRI: Magnetic resonance imaging
PARS: Percutaneous Achilles repair system
